# Influence of Glycerol and SISAL Microfiber Contents on the Thermal and Tensile Properties of Thermoplastic Starch Composites

**DOI:** 10.3390/polym15204141

**Published:** 2023-10-19

**Authors:** Mailson Batista de Vilhena, Rochelle Moraes Matos, Gilberto Sérgio da Silva Ramos Junior, Bruno Marques Viegas, Carlos Alberto Brito da Silva Junior, Emanuel Negrão Macedo, Marcos Vinícius da Silva Paula, José Antônio da Silva Souza, Verônica Scarpini Candido, Edinaldo José de Sousa Cunha

**Affiliations:** 1 Engineering of Natural Resources of the Amazon Program, Federal University of Pará—UFPA, Belem 66075-110, Brazil; mailson.vilhena@abaetetuba.ufpa.br (M.B.d.V.); enegrao@ufpa.br (E.N.M.); jass@ufpa.br (J.A.d.S.S.); 2 Faculty of Materials Engineering, Federal University of Pará-UFPA, Ananindeua 67130-660, Brazil; rochelle.ufpa@gmail.com (R.M.M.); cunhaed@ufpa.br (E.J.d.S.C.); 3 Materials Science and Engineering Program, Federal University of Pará—UFPA, Ananindeua 67130-660, Brazil; gilbertoramosjr26@outlook.com (G.S.d.S.R.J.); cabsjr@ufpa.br (C.A.B.d.S.J.); mpaula@ufpa.br (M.V.d.S.P.); 4 Faculty of Biotechnology, Federal University of Pará—UFPA, Belem 66075-110, Brazil; bviegas@ufpa.br

**Keywords:** glycerol, microfibers, composites, corn starch

## Abstract

The increasing use of petroleum plastics has caused environmental damage due to the degradation time of these materials. An alternative to petroleum plastics could be thermoplastic starch (TPS). However, thermoplastic starch does not exhibit satisfactory tensile properties. The mechanical properties of thermoplastic starch can be improved by adding sisal microfibers. Thus, the objective of this study was to evaluate the influence of different levels of glycerol and sisal microfibers on the thermal and tensile properties of thermoplastic corn starch composites. The microfibers were obtained via mechanical treatment followed by chemical treatment (alkaline treatment and bleaching). The films were obtained by the casting method using commercial corn starch and glycerol as a plasticizing agent, reinforced with sisal microfibers. Fourier transform infrared spectroscopy (FTIR) results revealed that the addition of microfibers did not change the chemical structure of the TPS matrix. The films from the samples with 18% glycerol and 10% microfibers had the highest value for the maximum tension, equal to 4.78 MPa. The thermal decomposition profile of TPS was not altered by the addition of microfibers. Our findings demonstrated the profound influence of glycerol and microfiber contents on the tensile properties of thermoplastic starch composites.

## 1. Introduction

The intense disposal of plastics from petroleum has been causing serious damage to the environment; on the other hand, biodegradable materials have received academic and industrial interest as an alternative to petroleum-derived plastics [1,2,3,4,5]. Biopolymers are biodegradable materials obtained from natural resources and renewables that are good candidates to replace petroleum-based plastics [6,7]. Biopolymers have advantages such as high availability, biodegradability, and low cost [8]. Among biopolymers, starch-derived polymers have received attention because they are cheap, abundant, and biodegradable [9,10,11].

Starch can be converted into thermoplastic materials in the presence of plasticizers such as water and polyols with low molar mass, but its application is still limited mainly due to its low mechanical performance compared to other materials [12,13]. Starch is a polysaccharide present in various plant sources, such as cereals, roots, and tubers, as well as extracted from fruits and vegetables [14]. For commercial business purposes, starch extraction is restricted to cereals and tubers. Starch is found in plants in the form of granules, where the starch has a degree of molecular organization, giving the starch a partially crystalline character [15]. Starch crystallinity is attributed to the presence of amylopectin, while amylose favors the formation of the amorphous structure of the material [16,17]. Thermoplastic materials derived from starch can be obtained after their granular structure (semicrystalline) is destroyed to produce a homogeneous polymeric matrix with an amorphous character [18]. The phenomena that destroy the organization of starch granules are gelatinization and melting [19]. Starch granules in contact with cold water swell slightly (10 to 20%) due to diffusion and absorption of water in the amorphous regions, but this occurs reversibly. However, the heated granules swell irreversibly, which characterizes the process of gelatinization. During the gelatinization process, there is a loss of the structural organization of the starch with the fusion of crystals [20].

Thermoplastic materials derived from starch are obtained by plastification, where a plasticizer additive is used to obtain thermoplastic starch [21]. Plasticizers are low-molar-mass substances used as additives in polymers [22]. The main function of the plasticizer is to improve flexibility by reducing the stress of deformation, hardness, density, viscosity, and electrostatic charge while at the same time increasing fracture resistance [22]. Plasticization mechanisms have been widely investigated, and the main phenomena are related to the lubricating effect and the free volume that the plasticizer provides. The plasticizing agents generally used to obtain thermoplastic starch are water, polyols, and amides [23,24].

The mechanical performance of thermoplastic starch can be improved by adding fillers [25,26]. Natural fibers can be used as fillers in polymeric materials. Vegetable fibers are renewable materials with positive characteristics such as wide availability, less abrasive character compared to synthetic fibers, biodegradability, low production cost, and low density, among others [27]. Sisal (*Agave sisalana*) is a plant native to Mexico; it is the main vegetable fiber produced in the world, corresponding to about 70% of the commercial production of all fibers of this type [28]. Sisal plants had a good adaptation in the Northeast region of Brazil, which is currently the largest producer and exporter of sisal fibers in the world [29]. Among the main applications of these fibers, it can be emphasized the automobile sector, the manufacture of ropes, twine, maritime cables, carpets, bags, and the production of high-strength kraft paper, among others [30]. 

Sisal fibers can be used as a dispersed phase in composites, aiming to reduce hygroscopicity and/or increase the interaction capacity between the fiber and matrix. Several methods can be used to achieve this. These methods can be chemical, physical, or biological, where the chemical treatment called alkaline treatment is highly used, probably due to its low cost and easy execution [31,32]. In addition, the alkaline treatment produces materials with good properties [31]. One of the main objectives of the alkaline treatment is the cellulosic extraction of natural fibers [33]. This extraction is usually carried out in two stages. The first, called pre-treatment, consists of a treatment before the isolation method associated with the removal of non-cellulosic components, resulting in purified fibers [31]. The second stage consists of extracting the cellulosic material in the form of microfibrils [34]. After alkaline treatment, the fibers are usually bleached with sodium hypochlorite [35]. The alkaline treatment, also called mercerization, is a process in which the fibers are treated chemically to remove lignin, pectin, and other substances such as waxes and natural oils that coat the surface of the fiber’s cell wall [36]. An effective alkaline concentration depends on the type of fiber; however, concentrations in the range of 4–5% are considered ideal for improving the mechanical properties of the fibers [36].

In this investigation, sisal fibers were previously mechanically treated with subsequent alkaline treatment with sodium hydroxide solution (NaOH) and bleaching with sodium hypochlorite (NaClO) to obtain sisal microfibers. Corn starch composites with different microfiber and glycerol contents were obtained by the solvent casting method and evaluated by techniques such as Fourier transform infrared spectroscopy (FTIR), thermogravimetric analysis (TGA), and scanning electron microscopy (SEM), as well as their mechanical properties. Thus, the main proposal of this investigation was to investigate the effect of adding different levels of glycerol and sisal microfibers on the thermal and tensile properties of thermoplastic starch composites. Our results showed improvement in the tensile properties without causing major changes in the chemical identity of the polymeric matrix.

## 2. Materials and Methods

### 2.1. Materials 

Corn starch was purchased from Maizena^®^ (Pernambuco, Brazil) and glycerol 80% was purchased from Pharmapele (Pará, Brazil). Sodium hydroxide and sodium hypochlorite were supplied from Dinâmica (Pará, Brazil) and Ypê (Pará, Brazil), respectively.

### 2.2. Preparation of Sisal Microfibers

The sisal fibers were ground in a knife mill TRF 600, reaching an average size of 1 cm in length. Then, the fibers were mechanically defibrillated using a blender and dried in an oven with air circulation at 35 °C for 24 h. After drying, they were ground again in a Marconi knife Willey mill model MA048, reaching a size of approximately 0.50 mm in length. The ground fibers were placed in a solution of 5% (*w*/*v*) NaOH for 1 h at 80 °C (in a water bath), in the proportion of 1 g of fiber to 20 mL of solution under mechanical agitation. After cooling, the material was vacuum filtered and washed with distilled water until a neutral pH was reached. After this step, the fibers were dried in an oven with air circulation at 35 °C for 24 h. The fibers treated with NaOH solution were immersed in NaClO 1% (*v*/*v*) in the concentration of 1 g of fiber to 20 mL of solution at 65 °C with stirring mechanics for 1 h. After that, the fibers were washed with distilled water to remove excess NaClO, filtered under vacuum, and dried in an oven with air circulation at 35 °C for 24 h.

### 2.3. Preparation of Composites

**Figure 1:**
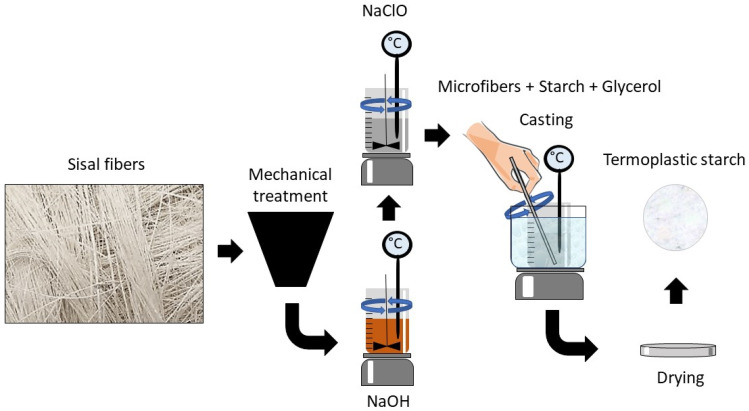
Schematic diagram of composite film preparation.

The thermoplastic starch was processed via solvent casting; a filmogenic solution was prepared by corn starch and distilled water in a proportion of 1:20 *w*/*v*, that is, 1 g of starch and 20 mL of distilled water were added. In addition to water, glycerol was used as a plasticizing agent in mass fractions of 18, 28, and 36% of the starch mass, which was homogenized, heated to 85 °C, and manually stirred until the gel point. The composites were obtained for the three glycerol contents with treated fiber contents equal to 5% and 10% of the starch mass. The solution was placed in silicone molds (18 × 19 cm) and dehydrated in an oven with air circulation (around 35 °C) for 24 h. The methodology used to obtain the films is summarized in Figure 1. The films obtained in this investigation are described in Table 1.

### 2.4. Fourier Transform Infrared Spectroscopy

Absorption spectra in the infrared region were acquired by attenuated total reflectance at room temperature using a Perkin Elmer FT-IR/FT-NIR spectrophotometer, Spectrum 400 Bruker. Spectra were obtained with a resolution of 8 cm^−1^, 100 scans, and a selection of waves situated between 4000 and 400 cm^−1^.

### 2.5. Thermogravimetric Analysis

The TGA curves for the films were acquired with a NEXTA STA 300 instrument under an inert nitrogen atmosphere (100 mL·min^−1^) in a range from room temperature to 800 °C at a rate of 10 °C·min^−1^.

### 2.6. X-ray Diffraction

The X-ray diffraction equipment used in our research experiments was the X-ray diffractometer model D8 ADVANCE, from Bruker, with goniometer (Theta\Theta) and Cu anode ceramic X-ray tube (Kα1 = 1.540598 Å), model 10190376. All measurements were made at room temperature with voltage and current at 40 kV and 40 mA, respectively. Data were collected in a range from 5 to 60 degrees, with angular intervals between experimental points of 0.02 degrees and a detection time of 1 second for each measurement point.

### 2.7. Scanning Electron Microscopy

SEM images were obtained using a scanning electron microscope (Hitachi TM3000) with an accelerating voltage of 5 kV. Surface images of the samples were performed at different magnifications. 

Samples of sisal fibers (before and after chemical treatment), plasticized starch films (with different glycerol contents), and composites (fracture region) were adhered to carbon tape on an aluminum support. After this step, the samples were inserted directly into the scanning electron microscope for image acquisition.

### 2.8. Tensile Properties

The tensile properties of TPS and composite films made with different contents of microfibers and glycerol were determined according to the ASTM D882-02 standard [37] in a universal mechanical testing machine, INTERMETRIC *im*50 (São Paulo, Brazil). The movies were cut to dimensions of 75 × 25 mm and tested at a pulling speed of 5 mm/min. The evaluated properties were tensile strength (σ), the modulus of elasticity (ε), and elongation at maximum strength (E). Five replicates were performed for each film, and the mean value was determined. Significant statistical variations were investigated by Duncan’s test.

## 3. Results and Discussion

### 3.1. Fourier Transform Infrared Spectroscopy

**Figure 2:**
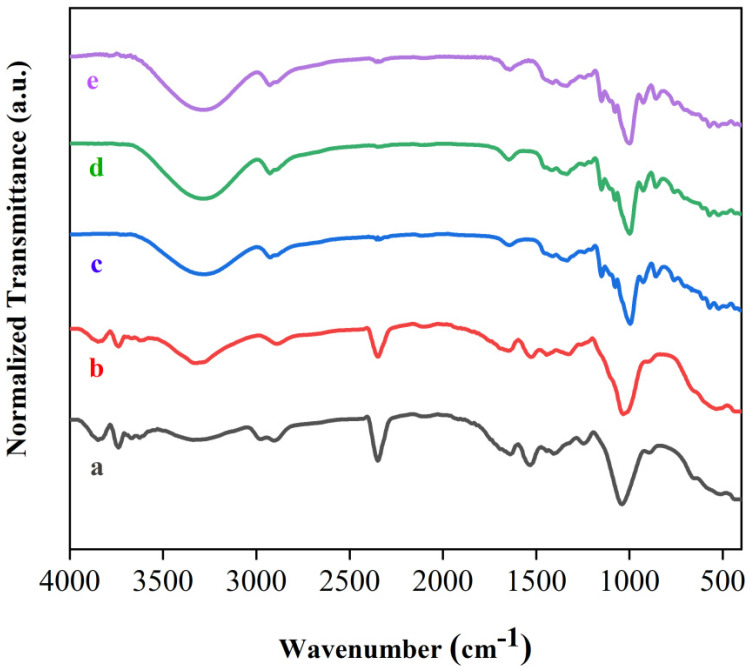
FTIR spectra of (a) fibers without treatment; (b) fibers with alkaline treatment and bleaching; (c) TPS-1; (d) TPS-2; (e) TPS-3.

FTIR spectroscopy was employed to access the vibrational modes of samples. Figure 2 shows the spectra for fibers without treatment, fibers with alkaline treatment and bleaching, and TPS films with the three amounts of glycerol. The FTIR spectrum for the untreated fibers revealed the presence of a band around 3330 cm^−1^, which occurs due to the presence of OH groups of polysaccharides [38]. The bands observed in the range from 2990 to 2820 cm^−1^ are attributed to C-H bonds that exist in lignin, hemicellulose, and cellulose [39]. The band observed at 1640 cm^−1^ is described as the presence of water in the fibers [40]. The band around 1438 cm^−1^ is described as vibrations of C-H bonds in cellulose [40]. The band at 1250 cm^−1^ is related to the C-O stretching of hemicellulose and aryl–alkyl ether from lignin [40]. On the other hand, chemically treated fibers exhibited small changes in the FTIR spectrum compared to untreated fibers. The bands at 2990 and 1250 cm^−1^ were almost not observed in fibers treated with NaOH solution, which is indicative of the elimination of a large part of lignin and hemicellulose [40], which was corroborated by the results of SEM images (Section 3.3). Our results are in good agreement with the literature and indicate that the alkaline treatment was able to isolate the cellulose from the sisal fibers [41].

TPS films exhibited very similar spectral behavior. In Figure 2, a band around 3290 cm^−1^ was observed for the three TPS films; these bands were associated with O-H stretching vibrations from starch and glycerol [42,43,44]. The bands displayed around 2929 and 2890 cm^−1^ were attributed to C-H bonds [45,46]. In Figure 2, bands were also observed in the region 1630–1650 cm^−1^. These findings were attributed to the absorbed water in the TPS films [47]. The vibrational modes observed at 999 cm^−1^ are associated with the stretching of the C-O bond, which is present in the glucose ring [48,49]. The bands at 1151 cm^−1^ and 1077 cm^−1^ were assigned to the C-O-C and C-O-H groups [50,51]. These results are in good agreement with Zhang et al. [12], Lai et al. [25], and Yang et al. [4]. Our results indicated that the chemical structure of TPS was not modified for the three levels of glycerol used for starch plastification.

**Figure 3:**
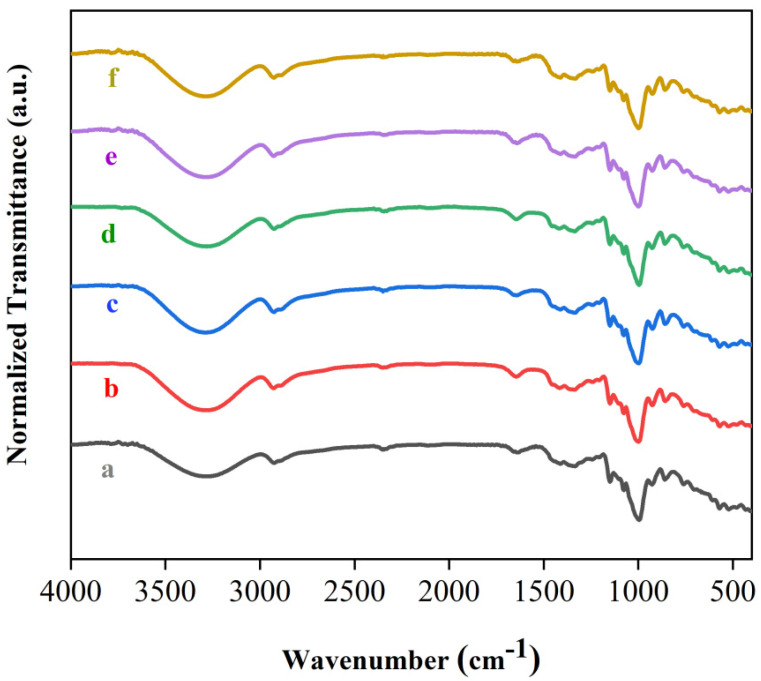
FTIR spectra of (a) CS-1; (b) CS-2; (c) CS-3; (d) CS-4; (e) CS-5; (f) CS-6.

Figure 3 shows the spectra for the composite films. The composite films showed the same spectral behavior obtained for the TPS films, with marginal variations. In the spectra for the composites, it is observed that the band attributed to the stretching of the O-H group was slightly shifted to higher values of wavenumbers; these shifts were due to the formation of hydrogen bonds between the fibers and the TPS matrix [52]. These shifts to higher values of wavenumbers for O-H groups were observed after the addition of coir fibers in the cassava starch matrix, with fiber contents ranging from 5 to 10% [53]. Prachayawarakorn et al., via compressing molding, obtained cassava starch composites with jute and kapok fibers and also observed marginal variations in the peak positions of the FTIR spectra of the composites in relation to the plasticized cassava starch matrix [54]. Syafri et al. reported that composites formed by plasticized starch from bengkuang and nanocellulose fibers from water hyacinth also exhibited a shift towards higher wavenumber values for the stretching of the O-H group [52]. Our findings demonstrated that the addition of natural fibers did not change the chemical structure of the TPS matrix.

### 3.2. Thermogravimetric Analysis

**Figure 4:**
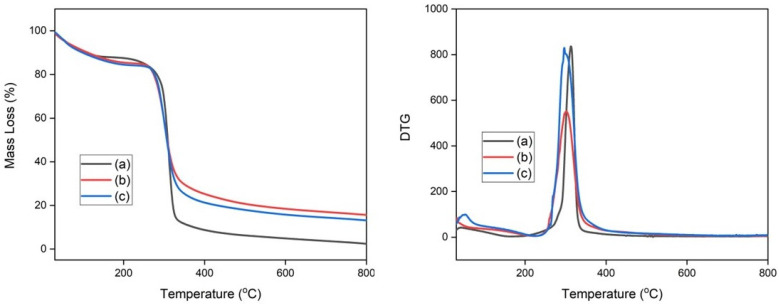
(**Left**) TGA curves of (a) TPS-1, (b) TPS-2, and (c) TPS-3; (**right**) DTG curves of (a) TPS-1, (b) TPS-2, and (c) TPS-3.

The thermal stability of TPS films and composite films was evaluated through thermogravimetric analysis. TGA curves were acquired in an inert nitrogen atmosphere in the interval between room temperature and 800 °C. Figure 4 shows the TGA and its derivative (DTG) curves for TPS films with different glycerol contents used for corn starch plastification. Figure 4 reveals that there are two mass loss events for the TPS films. The first event that occurs between 60 and 200 °C has been described as a loss of glycerol and adsorbed or weakly bound water in TPS films [4]. The second event in the range of 230 to 500 °C was reported as starch decomposition [55]. Table 2 presents the temperatures at which mass losses of 5% (T_5_), 10% (T_10_), 50% (T_50_), T_max_, and residual mass occur at 800 °C. The increase in the glycerol content decreased the thermal stability of the TPS films, as seen in Figure 3 and the values of T_5_, T_10_, and T_50_ in Table 2. This occurs because the glycerol molecules act by reducing the strong interactions between the starch chains [51]. 

These results obtained in our investigation are in good agreement with those reported by Hafila et al. [56], Hazrati et al. [57], and Florencia et al_._ [58]. Tarique et al. investigated the plastification of arrowroot starch with glycerol, with glycerol contents equal to 15%, 30%, and 45%. They also observed that increasing the glycerol content decreased the stability of plasticized arrowroot starch films; this behavior was attributed to the decrease in intermolecular forces between the starch chains after the addition of glycerol [51]. Wang et al. evaluated the plastification of corn starch with a polymeric ionic liquid and reported through differential scanning calorimetry tests that the addition of larger amounts of the polymeric ionic liquid decreased the glass transition temperature for the plasticized starch. This result was attributed to the interactions between the ionic liquid and the starch chains, where the ionic liquid acted to reduce the strong interactions between the starch chains [18].

**Figure 5:**
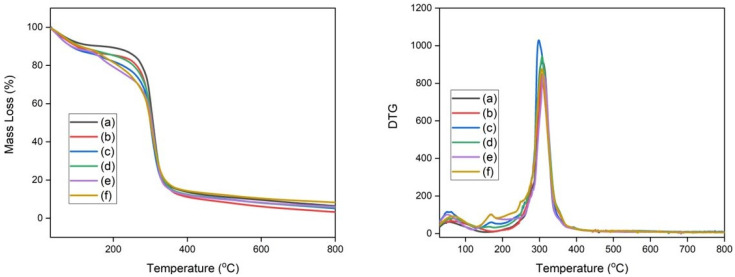
(**Left**) TGA curves of (a) CS-1, (b) CS-2, (c) CS-3, (d) CS-4, (e) CS-5, and (f) CS-6; (**right**) DTG curves of (a) CS-1, (b) CS-2, (c) CS-3, (d) CS-4, (e) CS-5, and (f) CS-6.

Figure 5 displays the TG and DTG curves for the composite films. The composites exhibited a thermal decomposition profile similar to the profile exhibited by TPS films plasticized with different glycerol contents, as shown in Figure 3 and Figure 4 and the values of T_5_, T_10_, T_50_, and T_max_ (Table 2). The first event between 130 and 230 °C was described as a loss of water and glycerol. The event between 240 and 480 °C was attributed to starch and cellulose decomposition. The samples CS-5 and CS-6 exhibited a more pronounced mass loss in the interval between 130 and 300 °C. This characteristic of thermal decomposition has been attributed to three factors: (a) the elimination of glycerol, which has a higher content in these samples; (b) the glycerol also acts by reducing the interaction between the starch chains associated with it; and (c) thermal decomposition of cellulose [4,38,51].

Prachayawarakorn et al. evaluated the effect of adding kapok fibers on the thermal properties of their composites with cassava starch matrix plasticized with glycerol. They reported that the degradation onset temperature decreased with increasing fiber content; this finding was associated with the hydrophobic character of the fibers. They also investigated the thermal properties of composites of thermoplastic cassava starch with jute fibers, but they did not observe a significant difference in the degradation onset temperature between the samples of starch and composites [54]. Syafri et al. evaluated the effect of sonication time on the thermal properties of nanocomposites formed by plasticizing bengkuang starch with nanocellulose obtained from water hyacinth [52]. They observed two main mass loss events attributed to moisture removal and further degradation of cellulose and starch, respectively. In addition, they also reported that non-sonicated samples showed lower thermal stability, which was interpreted as low dispersion of fibers in the polymeric matrix. Our findings revealed that the addition of sisal microfibers did not cause major changes in the TPS thermal decomposition profile.

### 3.3. Scanning Electron Microscopy

**Figure 6:**
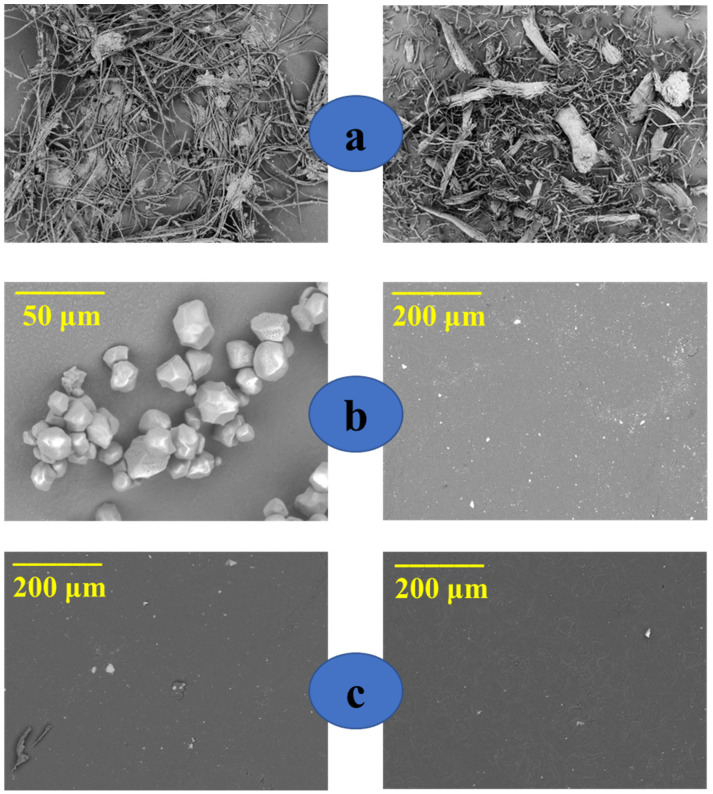
SEM images of (**a**) fibers without treatment (**left**), fibers with alkaline treatment and bleaching (**right**); (**b**) starch grains (**left**), TPS-1 (**right**); (**c**) TPS-2 (**left**), and TPS-3 (**right**).

The morphology of the fibers, surface, and fracture surface of the films were investigated by SEM image acquisition. The morphologies of the longitudinal surfaces of the fibers before and after bleaching are shown in Figure 6. Figure 6a (left) shows that in fibers without alkaline treatment, the bundles are joined by the non-fibrous components (lignin and hemicellulose), forming a structure of less exposed microfibrils. On the other hand, with the alkaline treatment and bleaching, most of these components around the beams have been removed Figure 6a (right) [59,60,61]. In addition, the SEM images demonstrated that the fibers after alkaline treatment have micrometric dimensions. Figure 6b (left) shows a SEM image for corn starch, where the presence of grains characteristic of corn starch is observed [62,63,64]. However, the images for the TPS films displayed in Figure 6b,c reveal the loss of a large part of the starch grains; this behavior occurred due to plastification with glycerol, which destroys the starch grains [18,51]. The images for the TPS films also revealed that the increase in the glycerol content produces a surface with fewer starch grains; the higher glycerol content provides a greater plastification effect on the starch, decreasing the amount of starch grains [42,65].

**Figure 7:**
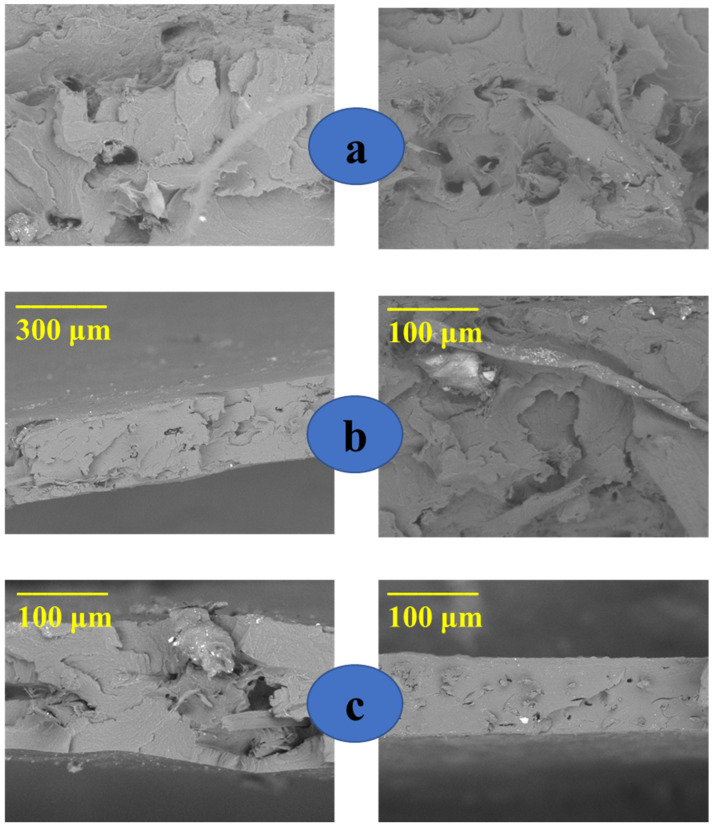
SEM images of the fracture surface of (**a**) CS-1 (**left**) and CS-2 (**right**); (**b**) CS-3 (**left**) and CS-4 (**right**); and (**c**) CS-5 (**left**) and CS-6 (**right**).

Figure 7 displays the fracture surface images of composite films, where the random dispersion of the microfibers in the polymeric matrix is observed for all samples evaluated [54].

### 3.4. X-ray Diffraction

**Figure 8:**
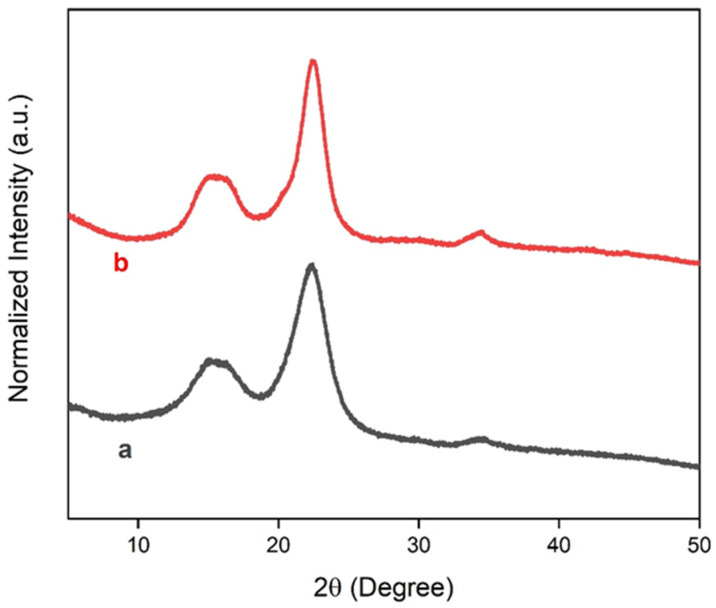
XRD of (a) fibers without treatment; (b) fibers with alkaline treatment and bleaching.

The crystalline behavior of microfibers and TPS films was determined by X-ray diffraction (XRD). Figure 8 presents the X-ray diffractograms of the fibers before and after the chemical treatment. The diffractograms showed peaks at 15.02° and 22.69°, which are characteristic of cellulose types I and II, respectively [66,67,68]. Treated fibers exhibited a narrow peak at 22.69° and an increase in peak intensity at 15.02° in relation to the untreated fiber, which is indicative of the increase in crystallinity of the fibers after the chemical treatment. The chemical treatment eliminates surface materials that have an amorphous character [69,70].

**Figure 9:**
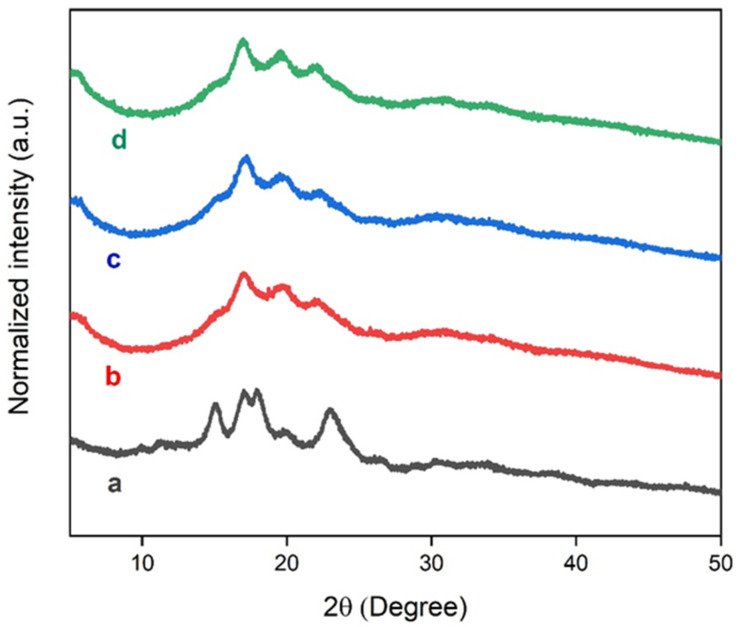
XRD of (a) starch grains; (b) TPS-1; (c) TPS-2; (d) TPS-3.

Figure 9 displays the diffractograms for starch and TPS films. The corn starch exhibited diffraction peaks at 15.1°, 17.1°, 17.97°, and 22.9°, which are characteristic of type A starch extracted from cereals [71,72,73,74]. Our XRD results for corn starch were corroborated by Amaral et al. [75]. The TPS films showed diffraction peaks at 17.02°, 19.7°, and 22.04°, where the peak at 17.02° represents amylose crystallization and amylopectin recrystallization. The peaks at 19.7° and 22° are attributed to amylose crystallization [76]. Our results indicate the formation of a semicrystalline material with the presence of non-plasticized grains, an amorphous material, and partial recrystallization of starch [76].

### 3.5. Tensile Properties

The effect of different levels of glycerol and microfibers on the tensile properties of the films was evaluated through the acquisition of stress–strain curves. Tensile strength (σ), elongation at maximum strength (E), and modulus of elasticity (ε) were evaluated. The mean values for σ, E, and ε for the TPS films are shown in Table 3. Significant statistical variations were determined by Duncan’s test with a 5% significance level.

For TPS films, a significant reduction in σ is observed with increasing glycerol content; films with the lowest glycerol content had a higher σ value equal to 3.25 MPa. The addition of 28 and 36% glycerol reduced the σ values to 2.50 and 1.30 MPa, respectively. This trend occurs because, in films with lower glycerol content, the strong hydrogen bonds formed between the starch chains predominate [51]. The increase in the glycerol content decreases the interactions between the starch chains, increasing the interactions between starch and glycerol [51,77,78]. Razavi et al. report that lower values of σ are obtained for films plasticized with glycerol compared to other plasticizer molecules; this behavior occurs due to the small size of the glycerol molecule [79]. On the other hand, for E, a significant increase was observed for TPS-2 and TPS-3 compared to TPS-1. This behavior can be explained by the decrease in intermolecular interactions between the starch matrix chains after the addition of glycerol [51].

Table 3 reveals a significant reduction for ε in TPS-2 and TPS-3 films compared to TPS-1 films. These results are expected since the plasticizer decreases the level of rigidity of the material and consequently increases its deformation. These results were observed due to the change in intermolecular interactions formed between the starch chains and the intermolecular interactions established between the starch chains and the plasticizer [51,80]. These findings are corroborated by investigations found in the literature [77,78,81,82].

Table 3 presents the σ, E, and ε results for the composite films. Table 3 reveals that the CS-2 film exhibits a higher mean value of σ in relation to the other films. This result may have occurred due to an association between the lower glycerol content and the presence of fibers. The lower glycerol content favors intermolecular interactions between the starch chains, and the addition of microfibers increases the resistance of the CS-2 films, increasing the σ value. The increase in the σ value for the CS-2 sample with 10% of microfibers also indicates a good dispersion of the microfibers in the polymeric matrix, revealing that the chemical treatment used in this investigation promoted a good interaction and compatibility between fibers and the TPS matrix [83,84,85]. The CS-1 film exhibited the second highest value for σ among the composites; this was due to the presence of fibers and the lower glycerol content used to obtain the CS-1 film, as well as good compatibility for the composite components. The improvement in the value of σ for CS-1 and CS-2 can also be attributed to the chemical similarity between the components of the composites [54]. This fact is important because, with the similarity between the components of the composite, it is possible to transfer the tension imposed on the matrix to the sisal microfibers, improving the values of σ and ε. The other composite films showed lower values of σ, which was mainly attributed to the higher glycerol content in the films. Campos et al. reported that alkaline treatment promotes wettability and better fiber-matrix adhesions, allowing efficient tension transfer between matrix and fibers [86]. The alkaline treatment removes the impurities and promotes defibrillation, increasing the effective surface area and providing better mechanical performance in materials [41,87]. These findings were corroborated by Jumaidin et al., who observed an increase in the value of σ in composites of thermoplastic cassava starch with 1, 3, and 5% of cogon grass fiber [84]. Jumaidin et al. attributed the improvement in σ values in the composites formed by the thermoplastic sugar palm starch/agar (TPSA) blend with *Eucheuma cottonii* seaweed waste to the good chemical compatibility between the components of the composite [88]. Composites prepared by extrusion and formed by blends of polycaprolactone/corn starch with sisal fibers treated with sodium hydroxide and bleached with hydrogen peroxide also exhibited an improvement in their tensile properties [83]. However, CS-1 and CS-2 films showed a significant decrease in E compared to TPS-1, TPS-2, and TPS-3 films; this can be explained by the stiffness of the fibers and the low glycerol content used for the film preparation [54]. Table 3 shows that the average values for ε showed a similar trend to the average values for σ. The CS-1 and CS-2 films had a significant increase in ε when compared to the other films investigated in our study. This behavior was attributed to the lower content of glycerol and the presence of fibers. The decrease in the glycerol content favors the starch-starch interactions, which favors the increase in the value of ε. In addition, the presence and random dispersion of microfibers, as visualized by SEM images of the fracture surface of the composites, favor the interaction between them and the polymeric matrix, increasing the value of ε. The CS-1 and CS-2 films exhibited the highest values for σ and ε; however, they exhibited a low value for E, which can hinder applications in food packaging. Therefore, CS-3 films can be recommended for packaging applications because they have a good combination of the values of σ, ε, and E [54,89]. Our results showed that the different contents of glycerol and sisal microfibers had a primordial effect on the tensile properties of the evaluated composites.

## 4. Conclusions

In this investigation, different levels of glycerol and sisal microfibers were evaluated for the thermal and tensile properties of starch and sisal microfiber composites. Firstly, sisal microfibers were successfully obtained by combining mechanical treatment and alkaline treatment with sodium hydroxide and subsequent bleaching with sodium hypochlorite. The SEM images revealed that the fibers were in micrometric dimensions and without the presence of amorphous compounds, which was corroborated by the XRD and FTIR results. After this, TPS and TPS composite films with chemically treated sisal microfibers were satisfactorily obtained by the solvent casting method. FTIR spectra revealed the formation of hydrogen bonds between starch and sisal microfibers via a shift to higher wavenumber values for the OH-stretching vibrations in all composite films. The absorption spectra in the infrared region showed that the addition of sisal microfibers did not modify the polymer structure in the composite films. TGA results showed that the increase in glycerol content decreased the thermal stability of the TPS films. This was observed because the glycerol molecules act by reducing the strong interactions between the starch chains. The presence of two contents of sisal microfibers did not cause major changes in the thermal decomposition profile of starch plasticized with 18, 28, and 36% glycerol. The fibers were randomly dispersed in the TPS matrix, as shown in the SEM images of the fracture surface of the composites. XRD analyses demonstrated that the TPS films exhibited the characteristic profile of corn starch plasticized with glycerol. Our investigation showed that different glycerol contents influenced the tensile properties of TPS films. In general, the increase in glycerol content promoted a decrease in σ and ε for TPS films, which was attributed to a decrease in interactions between starch chains due to the presence of glycerol. For composite films, the effects of different glycerol and sisal microfiber contents had a significant influence on the tensile properties of the composites. The addition of sisal microfibers and 18% glycerol promoted a decrease in the deformation of the CS-1 and CS-2 films, which was attributed to the presence of the fibers and the low content of glycerol. CS-3 films exhibited the best combinations for the average values of σ, ε, and E, which indicates their use in food packaging. Our findings demonstrated that different contents of glycerol and sisal microfibers influenced the thermal and tensile properties of starch composite films with these two components. Our investigation revealed that sisal microfibers have the potential to act as reinforcement in thermoplastic matrix composites, which can be used in the food packaging sector.

## Figures and Tables

**Table 1:** Description of the composition of the films.

Film	Composition
TPS-1	Starch with 18% glycerol
TPS-2	Starch with 28% glycerol
TPS-3	Starch with 36% glycerol
CS-1	Starch with 18% glycerol + 5% treated fibers
CS-2	Starch with 18% glycerol + 10% treated fibers
CS-3	Starch with 28% glycerol + 5% treated fibers
CS-4	Starch with 28% glycerol + 10% treated fibers
CS-5	Starch with 36% glycerol + 5% treated fibers
CS-6	Starch with 36% glycerol + 10% treated fibers

**Table 2:** T_5_, T_10_, T_50_, T_max_, and residual mass for sample films.

Sample	T_5_ (°C)	T_10_ (°C)	T_50_ (°C)	T_max_(°C)	Residual Mass (%) at 800 °C
TPS-1	57	104	309	313	2.40
TPS-2	57	111	307	302	15.60
TPS-3	54	98	305	297	13.10
CS-1	69	172	307	307	6.43
CS-2	61	102	305	306	3.26
CS-3	55	86	300	298	5.12
CS-4	67	112	305	307	5.55
CS-5	54	87	304	309	5.97
CS-6	66	110	301	307	8.25

**Table 3:** Tensile strength (σ), elongation at maximum strength (E), and modulus of elasticity (ε) for films.

Sample	σ (MPa)	E (%)	ε (MPa)
TPS-1	3.25 ± 0.45 ^a^	9.87 ± 4.50 ^a,b^	93.77 ± 31.24 ^a^
TPS-2	2.50 ± 0.26 ^b^	22.13 ± 4.24 ^c^	27.50 ± 4.46 ^b^
TPS-3	1.30 ± 0.24 ^c^	16.40 ± 2.90 ^d^	16.97 ± 2.83 ^b^
CS-1	3.70 ± 0.87 ^a^	4.15 ± 0.55 ^e^	208.50 ± 29.68 ^c^
CS-2	4.78 ± 0.34 ^d^	3.12 ± 0.47 ^e^	267.17 ± 68.02 ^d^
CS-3	2.45 ± 0.57 ^b^	11.67 ± 4.19 ^b^	44.02 ± 11.42 ^b,e^
CS-4	2.44 ± 0.13 ^b^	6.60 ± 1.48 ^a,e^	74.26 ± 24.08 ^a,e^
CS-5	1.94 ± 0.15 ^b,e^	9.14 ± 1.10 ^a,b^	44.54 ± 8.57 ^b, e^
CS-6	1.67 ± 0.33 ^c,e^	6.90 ± 0.52 ^a,e^	44.04 ± 17.53 ^b,e^

Means with the same letter in a column have no statistically significant difference (Duncan’s test, *p* > 0.05).

## Data Availability

Not applicable.
